# Impact of COVID-19 on the performance of emerging market mutual funds: evidence from India

**DOI:** 10.1186/s43093-021-00081-w

**Published:** 2021-10-16

**Authors:** Muhammad Sali Maheen

**Affiliations:** 1 TKM College of Arts and Science, Kollam, 691005 Kerala India; 2 Department of Commerce, School of Business Management and Legal Studies, University of Kerala, Thiruvananthapuram, 695581 India

**Keywords:** Mutual funds, Fixed effects model, Sys-GMM, Instrumental variables, G20, C23

## Abstract

The purpose of this paper is to examine the widely believed beating capacity of actively managed funds during the market downturn. This popular hypothesis has been tested with the performance of Indian Equity Mutual Funds during the pandemic period. The conditional alphas are estimated using lagged instrumental variables with the fixed effect/LSDV estimator and the sys-GMM estimator in contrast to the OLS estimation from a sample of 1271 schemes for 5 months from 1st March 2020 to 31st July 2020. The study’s findings indicate that the actively managed Indian mutual fund co-moves with the market and does not possess the ability to beat the market. The major implication comes from the application of fixed effect and GMM estimators for the performance evaluation of Indian Mutual Funds’ during the crisis period, and it serves the investors in deciding the profitable investment opportunities.

## Highlights

- The paper examined the ability of actively managed funds to beat the market downturn
- The study applied Sys-GMM and LSDV/fixed effect models for estimating the performance alpha
- The actively managed Indian mutual funds do not possess the ability to outperform the market

## Introduction

COVID-19 carries unprecedented economic damage over natural disasters or nuclear war or climate change, or localised disasters [4]. It has impacted almost all the economy’s spheres, such as production, consumption, and accumulation. The financial markets and its included segments like equity, bond, and commodity markets (oil and gold) are severely affected by the globally declared pandemic’s vicious hand. This year, the IMF forecasted a global growth at − 4.9%, which is 1.9 per cent below the World Economic Outlook (WEO) forecast for April 2020 [36]. The same source cited a sharp decline of 4.5% for the Indian economy, a historic low rate, due to the pandemic impact of COVID-19. M. Nicolas Firzli, the Director of World Pension Council and the Member of the Advisory board at World Bank Global Infrastructure Facility, referred pandemic period as ‘the greater financial crises’ [46]. So far, a limited number of studies have addressed the issue empirically and focused on the angle of stock market performance, crude oil price fluctuation, and bit-coin return [2, 4, 23, 25, 38, 41, 44].

Mutual funds are the favourite avenue for risk-averse investors, and it was severely affected by recent health crises. The equity-oriented schemes have exhibited a negative return of about 25% [13], and Franklin Templeton has announced a historic decision to wind up six debt fund schemes amidst the COVID-19 pandemic [21]. Even though the industry has witnessed a sharp increase in the number of new investors [33]. It may be due to the common belief that the actively managed funds always beat the recession and generate a superior return to the investors [14, 17]. Pastor and Vorsatz [35] examined the widely accepted market outperform concept during crises. Consistent with the literature, the present study intended to examine the outperformance of actively managed Indian equity Mutual Funds during the COVID-19 crisis period.

COVID-19 is an infectious viral disease diagnosed at Wuhan (a Chinese city) in late December 2019. Soon it began to spread all over the world along with travellers from China. On 11 March 2020, the World Health Organization (WHO) declared it a global pandemic and issued an advisory to take preventive measures. Globally, there were 20,687,815 confirmed cases of COVID-19, including 750,400 deaths (WHO statistics as of 10:42 am CEST, 14 August 2020). During the initial phase of the outbreak, Europe followed China for reported cases of mortality. Later, the virus spread to the USA with a notorious velocity. India, Brazil, Russia, and the Middle East were seriously affected in the later phase of June 2020. It has impacted almost all the sectors of financial markets such as banks, insurance, stock markets, and mutual funds.

In India, the first COVID-19 case was reported on 30th January 2020. Till now, there are 6, 61,595 (26.88%) active cases; 1,751,555 (71.17%) recovery with a 1.95% fatality rate (as on 14-08-2020, MHFA). To curb the disease contagion, the Govt. of India declared a lockdown on 24th March 2020. It has led to the rise of unemployment in the economy, and almost all the sectors were affected by the vicious cycle of COVID-19. However, the history of Indian MF began in 1963 with the Unit Scheme of Unit Trust of India (UTI). Their monopoly was curtailed by giving operational permission to the public sector MF’s such as SBI Mutual Funds, Canara Bank Mutual Funds, and so on. Later, the industry was opened to the private sector and the foreign institutional investors. Presently, there are 43 asset management companies (AMC’s) in operation with an average Asset under management (AUM) of ₹27.12 Trillion (as of 31 July 2020).

Actively managed funds are commonly believed for their beating capacity during the market downturn [32]. The study here only considered 1271 equity schemes which are actively managed for a management fee. The managers commonly charge them for this widely believed beating capacity on account of their enhanced informativeness than the market. The present study aimed to evaluate the performance of emerging Indian mutual funds’ during the crisis period and tested for the superior return using asset pricing models’ conditional version. The study’s significant contribution comes in two ways: first, applying sys-GMM and LSDV models in measuring the mutual fund’s performance. The conditional alphas are estimated using the publicly available instrumental variables after conducting the Relevance and Endogeneity test, and the Hausman statistics support for cross-sectional effects are fixed. Second, the study applied panel data analysis in Indian Mutual Funds’ overall performance during the pandemic period. The results revealed that the emerging Indian market lacks the ability of superior return during the crisis period. The results of the study are useful to the investors for deciding the profitable investment opportunities.

Following the introductory session, the study organises the literature review session, data and methodology session, results and discussion session, and the concluding session.

## Literature review

The previous studies were mainly focused on the COVID-19 and its incidental impact on stock markets’ performance [4, 31, 38, 41, 44]. Ashraf [4] examined the impact of COVID-19 confirmed cases and deaths on the return of the stock markets of 64 countries from 22 January 2020 to 17 April 2020. He used the panel data analysis over the classical event study methodology as the pandemic evolves over a while rather than a particular point of time. The methodology is better to capture both cross-sectional, as well as time-series variation [7]. Waheed et al. [45] analysed the impact of COVID-19 on the KSE100 index using quantile-on-quantile estimations. The study was conducted for 2 months from 26 February to 17 April 2020. Capella-Blancard and Desroziers [9] studied how markets integrated publically available information about the COVID-19 pandemic and the subsequent lockdowns. They considered a panel of 74 countries from January to April 2020. Their study concluded that the market response largely depends on the post crises health policies of the Govt. rather than the pre-existing market conditions.

Sansa [41] studied the impact of COVID-19 on China’s financial markets and the USA from 1 March 2020, to 25 March 2020. She applied simple regression in Double Log and Semi Log-Linear Models and found that there is a serious impact on China and the USA’s stock markets. Ramelli and Wagner [38] examined the unprecedented impact of COVID-19 on the US stock market and compared the impact with the previous pandemics in 1918–19, 1957–58, and 1968. Topcu and Gulal [44] examined the impact of COVID-19 on emerging stock markets for 10 March –30 April 2020. The pandemic outbreak’s impact is higher in the emerging Asian markets than in the European markets, the policies of the govt matter a lot. Baker et al. [6] examined the impact of COVID-19 on the US market. They used text-based analysis for a large set of daily data from 1900 and found that the COVID-19 impacts substantially than Spanish flu. Cheema et al. [11] studied the impact of both the COVID-19 and the global financial crisis on the ten largest economies’ stock markets. During both, the crisis period, the US treasuries and the Swiss franc were acted as the safe heaven for investments. Al-awadhi et al. [1] investigated the impact of COVID-19 on the Chinese stock market and found a significant negative effect across the market.

Liu et al. [31] studied the coronavirus outbreak on 21 leading stock markets using the event study methodology. They found that the markets fell quickly after the outbreak. Further, they conducted panel fixed effect estimation using the abnormal returns and their results confirmed the pessimistic behaviour of investors. Galema et al. [22] applied sys-GMM for estimating the fund’s realised performance with the characteristics.

The manager decision regarding the choice of stock and the time at which they implement their choice is the major factor which contributes to the superior return; generally known as the stock-picking ability and the market timing ability [16, 26, 29]. The Jensen’s alpha is commonly used for measuring the stock-picking ability of managers [3, 5, 10, 15, 18, 19, 28]. A negative alpha represents poor stock selection, whereas a positive alpha is a sign of a better selection. Actively managed funds are declared for better informed than the market and can generate superior returns to the investors [14, 17]. The popular hypothesis formulated by Moskowitz [32] state that the ‘actively managed funds were outperformance during the market downturn’. The present pandemic period is most suitable for testing this popular hypothesis of ‘outperformance of funds during the crises period’ [35]. The large price dislocation resulting from the market disruption provides an opportunity for fund managers to generate market outperformance. It has evaluated with Capital Asset Pricing Model (CAPM) using the GMM estimation.

In light of the above work of literature, none of the studies have empirically tested the impact of COVID-19 on emerging Indian Mutual Funds. In this regard, the study aimed to evaluate the impact of COVID-19 on Indian MF’s using sys-GMM in addition to the fixed effect/LSDV estimation for a period of 5 months from March 1 to July 31 of 2020.

## Data and methodology

The study considered variables in daily frequencies, spanning over a period of 5 months from 1st March 2020 to 31st July 2020. Further, the entire sample period is subdivided into three subsamples: 01-03-2020 to 30-04-2020; 01-05-2020 to 31-05-2020; and 01-06-2020 to 31-07-2020.

Data for net asset values (NAV’s) are extracted from the website of Association of Mutual Funds in India (AMFI) for 1271 actively managed Indian equity mutual funds and converted into the daily return of portfolios ($R_{{p_{i} ,t}}$) (see Eq. 1). Likewise, the market returns are computed using the daily data of NSE Nifty. The index represents around 70% market capitalisation and, hence, used as the proxy for the market.

1 $$R_{{p_{i} ,t}} = {\raise0.7ex\hbox{${\left( {{\text{NAV}}_{{p_{i} ,t}} - {\text{NAV}}_{{p_{i} ,t - 1}} } \right)}$} \!\mathord{\left/ {\vphantom {{\left( {{\text{NAV}}_{{p_{i} ,t}} - {\text{NAV}}_{{p_{i} ,t - 1}} } \right)} {{\text{NAV}}_{{p_{i} ,t - 1}} }}}\right.\kern-\nulldelimiterspace} \!\lower0.7ex\hbox{${{\text{NAV}}_{{p_{i} ,t - 1}} }$}}$$

2 $$R_{m,t} = {\raise0.7ex\hbox{${\left( {{\text{IndV}}_{t} - {\text{IndV}}_{t - 1} } \right)}$} \!\mathord{\left/ {\vphantom {{\left( {{\text{IndV}}_{t} - {\text{IndV}}_{t - 1} } \right)} {{\text{IndV}}_{t - 1} }}}\right.\kern-\nulldelimiterspace} \!\lower0.7ex\hbox{${{\text{IndV}}_{t - 1} }$}}$$

IndV stands for the value of the market index *t*th day and *t *− 1th day, respectively. The details of schemes used for the analysis are presented in Table 1.

**Table 1:** Details of MF schemes

	Sample 1	Sample 2	Sample 3	Overall Sample
No. of Portfolio’s	1220	1219	1267	1271
No. of days in each samples	38	19	45	102
No. of observation	44,018	22,519	54,780	121,317

COVID-19 reported cases (${\mathbb{Z}}_{1}$), NSE Nifty dividend yield (${\mathbb{Z}}_{2}$), Oil price fluctuation (${\mathbb{Z}}_{3}$), Foreign exchange rates (${\mathbb{Z}}_{4}$), and Gold price fluctuations (${\mathbb{Z}}_{5}$) are taken as the instrumental variables and all the IV’s are in the demeaned form. The data for COVID-19 reported cases were retrieved from the website of ‘our world in data’ (https://ourworldindata.org/) and converted into the log form. All other variables are extracted from the investing database.

Sharpe [43] developed Capital Asset Pricing Model (CAPM) an equilibrium model for capturing the systematic risk using the market return over the risk-free return. He used single market factor to explain the overall performance of fund (see Eq. 3). Jensen [27] extended the model with an intercept term called alpha ($\alpha$). This term will capture manager’s ability to generate excess return. Now, the model becomes

3 $$R_{p} = R_{f} + \beta (R_{m} - R_{f} )$$

4 $$R_{p} - R_{f} = \alpha + \beta (R_{m} - R_{f} ) + \varepsilon_{i}$$

Here, $R_{p}$ stands for fund return; $R_{f}$ stands for the risk-free return; $\alpha ,\beta$ are the intercept and the slope of the equations.

Many papers acknowledged the use of instrumental variables to see the time varying nature of factor exposures. The initial category was to use conditional beta approach suggested by Ferson and Schadt [20]. The commonly used information variables such as interest rate, exchange rates, and dividend yield were used as the external instruments [5, 8, 28, 30, 39, 42]. Here, in this paper, lagged COVID-19 reported cases were used as an instrument to capture the performance of Indian Mutual Funds in this disrupted times. Some of the papers were attempted to make the alpha time variant and found a little evidence for alpha to exhibit the time variant behaviour [12]. Even though model captures the conditional performance better, they were prone to exhibit non-normality of residuals and leads to the over parameterisation [30].

Here, in this paper, the panel data estimation is proposed over the time series forecasting as they capture the movement of variable across time and space. The panel consists of large cross sections with the small time dimension (*N* > *T*), which will attract the conditions of GMM estimation. In this regard, the study used sys-GMM (Generalised Method of Momentum) estimation over the conventional pooled OLS estimations. Galema et al. [22] used sys-GMM proposed by Hansen [24] for estimating portfolio’s outperformance. Likewise, Roy and Shijin [40] applied IV-GMM for estimating the six factor models for US market.

### System-generalised method of moments (sys-GMM)

Standard IV estimator is a special case of generalised method of momentum with the following assumption.

The IV’s are exogenous to the error term denoted by $E\left( {{\rm Z}_{i}^{{}} u_{i} } \right) = 0.$ The *K* instruments give *K* moments,

5 $${\text{where}}\,\,\,\,g_{i} \left( {\hat{\beta }} \right) = {\rm Z}_{i}^{^{\prime}} \hat{u}_{i} = {\rm Z}_{i}^{^{\prime}} \left( {y_{i} - X_{i} \hat{\beta }} \right)$$

$$g_{i}$$ is a $$K \times 1$$ vector,

$$\begin{gathered} E(g_{i} (\beta )) = 0. \hfill \\ \hfill \\ \end{gathered}$$

Each *K* moment equations corresponds to a sample moment, and the form of the sample moments is as follows

6 $$\overline{g} \left( {\hat{\beta }} \right) = \frac{1}{n}\sum\limits_{i = 1}^{n} {g_{i} \left( {\hat{\beta }} \right) = } \frac{1}{n}\sum\limits_{i = 1}^{n} {Z^{\prime}} \left( {y_{i} - X_{i} \hat{\beta }} \right) = \frac{1}{n}Z^{\prime}\hat{u}$$

The very purpose of GMM is to choose an estimator for $\beta$, which solve $g_{i} \left( {\hat{\beta }} \right)$. If *K* moments equal to the *L* coefficient, in this case, we can have many equations. If *K* moments are unknown, then we can have *L* coefficient for $\hat{\beta }$. This GMM estimator is called IV estimator. If moments are higher than the coefficient, i.e., *K* > *L*, in this case, the estimation is not possible as we have many equations. In order to deal with this situation, create a weighing matrix *W* of order $K \times K$ and it is used for constructing quadratic form of moment conditions. This will result into the GMM objective function of

7 $$J\left( {\hat{\beta }} \right) = n\overline{g}\left( {\hat{\beta }} \right)^{\prime } W\overline{g}\left( {\hat{\beta }} \right)$$

The GMM estimator is created by using the following equations,

8 $$\frac{{\partial J\left( {\hat{\beta }} \right)}}{{\partial \hat{\beta }}} = 0$$

9 $$\hat{\beta }_{gmm} = \left( {X^{\prime}ZWZ^{\prime}X} \right)^{ - 1} X^{\prime}ZWZ^{\prime}y$$

### Conditional CAPM

In order to make the beta conditional on lagged information variable ${\mathbb{Z}}_{t - 1}$ the market factor $\beta (R_{m,i} - R_{f,i} )$ becomes the $\beta_{m} ({\mathbb{Z}}_{t - 1} )(R_{m,i} - R_{f,i} )$.

$$\begin{aligned} & {\text{Where}}\quad E({{\varepsilon_{i} } \mathord{\left/ {\vphantom {{\varepsilon_{i} } {{\mathbb{Z}}_{t - 1} ) = 0}}} \right. \kern-\nulldelimiterspace} {{\mathbb{Z}}_{t - 1} ) = 0}} \\ & {\text{and}}\quad E({{\varepsilon_{i} } \mathord{\left/ {\vphantom {{\varepsilon_{i} } {R_{m,i} - R_{f,i} ) = 0}}} \right. \kern-\nulldelimiterspace} {R_{m,i} - R_{f,i} ) = 0}} \\ \end{aligned}$$

the model becomes

10 $$\begin{aligned} R_{p,i} - R_{f,i} & = \,\alpha + \beta_{1} (R_{m,i} - R_{f,i} ) + \beta_{2} ({\mathbb{Z}}_{1,t - 1} )(R_{m,i} - R_{f,i} ) + \beta_{3} ({\mathbb{Z}}_{2,t - 1} )(R_{m,i} - R_{f,i} ) \\ & \quad + \beta_{4} ({\mathbb{Z}}_{3,t - 1} )(R_{m,i} - R_{f,i} ) + \beta_{5} ({\mathbb{Z}}_{4,t - 1} )(R_{m,i} - R_{f,i} ) + \beta_{6} ({\mathbb{Z}}_{5,t - 1} )(R_{m,i} - R_{f,i} ) + \varepsilon_{i} \\ \end{aligned}$$

${\mathbb{Z}}_{1to5}$ are the respective instrumental variables used for making the performance conditional on the one period lagged values. The study used the COVID-19 reported cases as an instrument to measure the conditional performance during the crises period. Further, the value of alpha is used as a measure to examine the performance of mutual funds. This is made possible through the OLS-fixed effect/LSDV estimation and the sys-GMM estimation.

### Proposed hypothesis

The value of conditional alpha measures the ability to generate superior returns [5]. The alpha close to zero means no superior return; managers may fall under or over-performance in other cases. The study’s hypothesis frames that the “actively managed funds outperform the market” [32]. Actively managed funds try to generate a superior return by beating the benchmarking indices. The Jensen’s alpha captures the value of over-performance. They are widely believed for their informativeness than the market and able to exhibit superior performance. In this study, the author wishes to measure overall performance using the unconventional methodology, i.e., fixed effect and sys-GMM estimator; than the micro-level performance using time series analysis. It can be seen from the corresponding p value of alpha, the intercept of both the system GMM estimator and the fixed effect estimator.

H1: Actively managed funds generate superior alpha, i.e., $\alpha > 0$.

Alpha’s for both the estimator is hypothesised as follows,

$$\begin{aligned} & H_{1a} :\alpha_{{\text{sys - gmm}}} > 0 \\ & H_{1b} :\alpha_{{\text{fixed - eff}}} > 0 \\ \end{aligned}$$

## Results and discussion

Descriptive statistics of the variables portfolio return and the excess market returns are shown in panel A of Table 2. The whole period statistics especially the mean and the median are reported with a negative return. This indicates an unfavourable impact of the crisis on the variables. The results further exhibit that the variables are negatively Skewed and Leptokurtic. The normality condition is violated as the *p* values are insignificant; for this, the Jarque–Berra statistics are estimated. Both the variables are positively correlated at 34% and the results are reported in the Panel B of Table 2.

**Table 2:** Table showing Descriptive Statistics of Variable and Cross correlation results

	$R_{p} - R_{f}$	$R_{m} - R_{f}$
*Panel A: Descriptive statistics of variables*		
Mean	− 0.060195	− 0.059684
Median	− 0.0589	− 0.0564
Std. Dev	0.023431	0.028072
Skewness	− 25.74997	− 1.258902
Kurtosis	1072.591	8.210543
Jarque–Bera	6.16E + 09	186,758.9
Probability	0	0
*Panel B: Pair wise correlation between variables*		
$R_{p} - R_{f}$	1	
$R_{m} - R_{f}$	0.3440594	1

*Source*: Computation by author

### Testing the relevance of instruments

Instruments validity is estimated using the relevance test proposed by Olea and Pflueger [34]. In this paper, I have applied the similar procedure as adopted by Roy and Shijin [40]. The explanatory variables are regressed with the instrumental variables and the resultant *F* statistics are used for measuring the weak instrument problem, i.e., if the value is less than 24, it indicates weak instruments or otherwise the instruments are robust.

11 $$R_{m} - R_{f} = \varphi + \gamma_{1} {\mathbb{Z}}_{1} + \gamma_{2} {\mathbb{Z}}_{2} + \gamma_{3} {\mathbb{Z}}_{3} + \gamma_{4} {\mathbb{Z}}_{4} + \gamma_{5} {\mathbb{Z}}_{5} + \nu_{i}$$

The results are reported in Table 3, as follows. The *t* values corresponding to the instruments are valid. ${\mathbb{Z}}_{1} ,{\mathbb{Z}}_{3}$ are positive coefficients, whereas the ${\mathbb{Z}}_{2} ,{\mathbb{Z}}_{4} ,{\mathbb{Z}}_{5}$ are negative coefficients. Relevance *F* indicates a favourable value which is higher than the standard value prescribed by the Olea and Pflueger [34]. The test accepts the overall validity of the instruments used for measuring the conditional performance and the GMM estimation and the instruments are robust.

**Table 3:** Result of relevance test

Variable	Coefficient	*t* statistic	*p* values
$\varphi$	− 0.059646	− 38,031.4	0
${\mathbb{Z}}_{1}$	0.000725	655.2984	0
${\mathbb{Z}}_{2}$	0.998269	15,761.56	0
${\mathbb{Z}}_{3}$	0.000298	64.19975	0
${\mathbb{Z}}_{4}$	0.011038	29.89703	0
${\mathbb{Z}}_{5}$	0.00106	10.29799	0
*F* statistic	62,834,864		
Prob. (*F* statistic)	0		

*Source*: Computation by author

### Exogeneity test

In order test the exogeneity of instruments (i.e., independence between error term and the instrumental variables) here, in this paper, I have regressed the instruments with the error term obtained by the unconditional version CAPM (see Eq. 4) [40]. As in Racicot and Rentz [37], “the coefficients of this regression disaggregate the effect of each regressor with the error term”.

12 $$\hat{\varepsilon }_{i} = \phi_{1} + \phi_{2} {\mathbb{Z}}_{1} + \phi_{3} {\mathbb{Z}}_{2} + \phi_{4} {\mathbb{Z}}_{3} + \phi_{5} {\mathbb{Z}}_{4} + \phi_{6} {\mathbb{Z}}_{5} + \xi_{1}$$

Here, $\hat{\varepsilon }_{i}$ stands for the estimated residual of unconditional CAPM. The resultant ($\phi_{{{\text{1to6}}}}$) will be the equivalent to coefficient of partial correlation. Result is given in Table 4, as follows.

**Table 4:** Results of Exogeneity Test and Hausman Test

Variable	$\phi_{1}$	${\mathbb{Z}}_{1}$	${\mathbb{Z}}_{2}$	${\mathbb{Z}}_{3}$	${\mathbb{Z}}_{4}$	${\mathbb{Z}}_{5}$	*F* stat	Pro	
*Panel A: Results of exogeneity test*									
Coef:	− 7.64E−05	0.00059	− 0.00375	− 0.0006	0.02743	− 0.0033	33.077	0	
*t* statistic	− 1.17434	12.5560	− 1.43421	− 3.2418	1.79925	− 0.7814			
*p* values	0.2403	0	0.1515^a,b^	0.0012	0.072^a,b^	0.4346^a,b^			

$\chi^{2}$ statistic	Chi-Sq. d.f	Prob
*Panel B—Hausman test (summary results)*		
13,312.510070	6	0.0000

*Source*: Computation by author

a, b are, respectively, 5% and 1% significance level

The coefficients of all the instruments are close to zero, and the *t* values corresponding to ${\mathbb{Z}}_{2} ,{\mathbb{Z}}_{4} \;{\text{and}}\;{\mathbb{Z}}_{5}$ are indeed exogenous, and the variables such as ${\mathbb{Z}}_{1} ,{\mathbb{Z}}_{3}$ are endogenous. The results of F-statistics and the corresponding *p* values are altogether accepted the overall exogeneity of the instruments. The instruments are strongly correlated with the potentially endogenous variable and genuinely exogenous to the model from this analysis.

Further, the study conducted the Hausman test for determining the individual effects are fixed or random. The $\chi^{2}$ statistic is found to be in favour of a fixed effect. In this regard, the performances of the funds are estimated using the fixed effect model and the sys-GMM. The results are shown in panel B of Table 4.

The fund specific performance ($\alpha$) during all subsample periods is found to be negative and significant. It is clear cut evidence for the adverse impact of the COVID-19 crisis on India’s mutual fund performance. The negative return is slightly higher in the LSDV model than GMM estimation except during the first sample period. The $\overline{R}^{2}$ results are slightly higher in GMM than fixed effect/LSDV results during the first and second sample periods; otherwise, $\overline{R}^{2}$ is found to be high in the LSDV model. The DW statistic measures the autocorrelation in residuals. The autocorrelation is positive in every sample period except slightly negative in first sample period. The market risk factor $\beta$ is higher in the fixed effect model during the first sample period, and in all other cases, $\beta$ extracted from GMM is found to be superior. The F stat of the fixed effect/LSDV models is found to be satisfactory, whereas the GMM exhibited over-identification of instruments with a significantly higher *J* value, which indicates trouble in using the instruments together. All the instruments are valid for the OLS-fixed effect model.

Further, the instruments ${\mathbb{Z}}_{1\& 3}$ are removed from fixed effect and GMM estimation, and it was found that there will not have any significant difference in the results of the estimate except for the J-stat of GMM. It can be inferred that the market is highly volatile at the time of recession or crisis period. The celebrated theory of high return for bearing high risk is violated.

Here, in this study, the popular hypothesis of superior $\alpha$ during the crisis period is violated irrespective of the sample period and the type of models. All the sample periods exhibited a negative fund performance and co-moves with the market downturn than the expected superior performance. The emerging market mutual fund does not hold the ability to beat the market to generate superior $\alpha$ during the recession period. The results are exhibited in Table 5 as follows.

**Table 5:** Regression results of LSDV model and GMM with instruments

	FIXED effect model					sys-GMM			
		$\alpha$	$\beta$	$\overline{R}^{2}$	DW	$\alpha$	$\beta$	$\overline{R}^{2}$	DW
*Regression results using all the five instruments*									
Sample 1	coef	− 0.0421	0.3261	0.3285	2.2803	− 0.0444	0.2876	0.3296	2.2537
		− *223.17*	*72.893*			− *152.82*	*66.084*		
Sample 2	coef	− 0.0452	0.2572	0.2412	1.7431	− 0.0445	0.2577	0.267	1.7052
		− *160.54*	*52.059*			− *173.61*	*62.594*		
Sample 3	coef	− 0.0453	0.2472	0.231	1.352	− 0.0426	0.2816	0.0072	1.022
		− *75.617*	*17.0406*			− *18.846*	*6.9031*		
Overall sample (Z1 to Z5)	coef	− 0.0432	0.28475	0.2716	1.6234	− 0.0409	0.32608	0.1257	1.34373
		− *248.49*	*100.83*			− *111.18*	*53.4034*		
	*F* stat		35.4966			*J* Stat		279.812	
	Prob(*F*)		0			*P* (*J* stat)		0	
*Regression results (after removing Z*_*1*_* and Z*_*3*_*)*									
Overall sample^*^ (reiterated)	coef	− 0.04161	0.313766	0.269382	1.631407	− 0.0483	0.201002	0.109798	1.364964
		− *276.677*	*132.7246*			− *75.1156*	*18.60871*		
	*F* stat		35.46262			*J* Stat		92.20145	
	*p* (*F* stat)		0			*P* (*J* stat)		0	

*Source*: Calculation by author

1. The italic fond represents the *t* stat of the variables

2. *J* stat represents the Hansen’s over-identification test of instruments in GMM

3. DW stands for the Durbin Wattson statistics for the autocorrelation of residuals

*Sample 1–3 are estimated using ${\mathbb{Z}}_{{{\text{1to5}}}}$ and ${\mathbb{Z}}_{1\& 3}$ are omitted in the overall sample estimation

## Conclusion

The study intended to analyse the performance of actively managed Indian Mutual funds during the COVID-19 pandemic period. The sample consists of 1271 actively managed mutual fund schemes over a period of 5 months from March to July 2020 in the daily data frequency. The fund performance is estimated using the conditional version of CAPM and applied the lagged form of IV’s such as COVID-19 reported cases (${\mathbb{Z}}_{1}$), NSE Nifty dividend yield (${\mathbb{Z}}_{2}$), Oil price fluctuation (${\mathbb{Z}}_{3}$), Foreign exchange rates (${\mathbb{Z}}_{4}$), and Gold price fluctuations (${\mathbb{Z}}_{5}$). Further, the instruments are analysed for relevance and the exogeneity. The results indicate that the instruments are jointly valid with a weak result on ${\mathbb{Z}}_{1}$ and ${\mathbb{Z}}_{3}$. The Hausman test results indicate that the individual effect is fixed, hence applied the fixed effect model over the Panel Least Square estimation. The panel consists of a large cross section (*N*) with a short period (*T*); this eventually attracts for applying system GMM estimation.

The alpha’s (intercepts) are estimated using the fixed effect model, and the sys-GMM is negative; this indicates the inadequate beating capacity of Indian fund houses. The results indicate that the popular hypothesis of superior alpha during the crisis period is violated throughout the sample period. There is no significant difference for the results estimated using system GMM and fixed effect model. The findings may be useful to the investors and managers for deciding the investable universe’s overall performance. The study used a novel application to the panel data methodology for deciding the performance of emerging market mutual funds in the crisis period. Further, it can be extended with a performance comparison of active and passive funds or even compare the robust estimator for the fund house’s overall performance.

## Data Availability

The data are available on request.

## Abbreviations

AMC: Asset management company
AUM: Asset under management
CAPM: Capital asset pricing model
GMM: Generalised method of moment
LSDV: Least square dummy variable
MF: Mutual fund
